# Hereditary cancer syndromes in Latino populations: genetic characterization and surveillance guidelines

**DOI:** 10.1186/s13053-017-0063-z

**Published:** 2017-01-21

**Authors:** Marcia Cruz-Correa, Julyann Pérez-Mayoral, Julie Dutil, Miguel Echenique, Rafael Mosquera, Keila Rivera-Román, Sharee Umpierre, Segundo Rodriguez-Quilichini, Maria Gonzalez-Pons, Myrta I. Olivera, Sherly Pardo, on behalf of the Puerto Rico Clinical Cancer Genetics Consortia

**Affiliations:** 1 Department of Cancer Biology, University of Puerto Rico Comprehensive Cancer Center, San Juan, PR USA; 2 University of Puerto Rico Medical Sciences Campus, School of Medicine, San Juan, PR USA; 3 Ponce Health Sciences University, Ponce Research Institute, Ponce, PR USA; 4 Cancer Center, Auxilio Mutuo Hospital, San Juan, PR USA; 5 Puerto Rico Gastroenterology Association, San Juan, PR USA; 6 Department of Pediatrics, University of Puerto Rico School of Medicine, San Juan, PR USA; 7 Department of Pathology, University of Puerto Rico School of Medicine, San Juan, PR USA; 8 University of Puerto Rico Comprehensive Cancer Center, PMB711 Ave. De Diego 89 Ste. 105, San Juan, PR 00927-6346 USA

**Keywords:** Genetic testing, Genetic counseling, Germline mutations, Hereditary cancer, Hispanics

## Abstract

Hereditary cancer predisposition syndromes comprise approximately 10% of diagnosed cancers; however, familial forms are believed to account for up to 30% of some cancers. In Hispanics, the most commonly diagnosed hereditary cancers include colorectal cancer syndromes such as, Lynch Syndrome, Familial Adenomatous Polyposis, and hereditary breast and ovarian cancer syndromes. Although the incidence of hereditary cancers is low, patients diagnosed with hereditary cancer syndromes are at high-risk for developing secondary cancers. Furthermore, the productivity loss that occurs after cancer diagnosis in these high-risk patients has a negative socio-economic impact. This review summarizes the genetic basis, phenotype characteristics, and the National Comprehensive Cancer Network’s screening, testing, and surveillance guidelines for the leading hereditary cancer syndromes. The aim of this review is to promote a better understanding of cancer genetics and genetic testing in Hispanic patients.

## Background

### Hereditary cancer overview

According to the 2010 census, Hispanics comprise 16.3% of the population in the United States (US) [1]. By 2050, the Hispanic population is estimated to become 25% of the US population. Hispanics are a mixed group of individuals from across Latin America and the Caribbean and are defined as individuals that share a culture and language that is linked to the historic migrations that started from Spain that led to the discovery of the Americas. In the year 1492, Christopher Columbus crossed the Atlantic Ocean and reached the Americas. The arrival of Columbus to the Americas led to the start of the “Colonization of Americas”, where language, culture and religion was instituted by the Spaniards, but also other European countries such as Portugal. Spaniards introduced new diseases to the Americas leading to the death of most of the Indigenous population of the Americas. Due to the high mortality of Indigenous population, Africans (mostly from West Africa) were introduced in the Americas. Through these historic migrations, the Hispanic population was created. These populations had great levels of genetic and cultural admixture from the three major racial ancestral populations: Europeans, Africans, and Indigenous. However, the variability observed in Hispanic admixture, is concordant with the local history of the country of origin [2]. For instance, Hispanics from the Caribbean (including Puerto Ricans) have a higher proportions of West African compared to Hispanics from South America, which have higher European and Amerindian ancestry [2].

Cancer is a major public health problem and the leading cause of death among Hispanics worldwide [3]. Malignancies, such as breast and colorectal cancers, when diagnosed in individuals younger than 50 years, are likely caused by inherited mutations and often are aggressive leading to a worse prognosis [4]. With the availability of efficient screening methods for both breast and colorectal cancer, these cancers may be prevented or diagnosed at early, more treatable stages in Hispanics, minimizing the burden of these diseases in this population [5]. By identifying individuals at high-risk and establishing tailored early detection and preventive strategies, the economic productivity loss, morbidity, and mortality may be significantly reduced.

In this article, we provide a summary of the genetic basis for three of the leading cancers in Hispanics (breast, ovarian, and colorectal cancer), which have known hereditary contributions. We will discuss the clinical presentation of these cancers in Hispanics and how it differs from other populations. Furthermore, we will discuss the role of multigene testing in advancing genetic testing services in Latino populations.

### Hereditary breast cancer and ovarian cancer (HBOC)

Breast cancer (BC) represents approximately 29% of all cancer cases in US Hispanic women [3]. Ovarian cancer (OC) is the 8^th^ cancer with the highest incidence and the 5^th^ leading cause of cancer death among US Hispanic women [3]. Approximately, 5–10% of all BC are caused by mutations in highly penetrant susceptibility genes [6]. Mutations in the *BRCA1* and *BRCA2* genes account for approximately 25% of hereditary BC and 15% of all OC [7]. Women with mutations in these genes have up to an 87% lifetime risk of developing BC by age 70 and up to a 44% risk of OC [4, 8].

#### Mutation spectrum

In Hispanics, deleterious *BRCA* mutations have been found in approximately 25% of women with BC [9]. The majority of the studies reporting the prevalence of *BRCA* mutations in Hispanics have been performed on heterogeneous groups of Hispanic women from different countries, including: Mexico [9–11], Brazil [12–14], Costa Rica [15], Chile [16, 17], Argentina [18], Peru [19], Colombia [20, 21], Venezuela [22], Bahamas [23], Cuba [24], and Puerto Rico [25] (Table 1). Recently, *BRCA* mutations in Latin American countries was published, which detailed the mutations found and how the prevalence changed from country to country [26]. All of these studies have shown that the *BRCA* mutation spectrum varies according to the country of origin. For example, women from Argentina, Mexico, and Brazil have a higher prevalence of *BRCA1* mutations [13, 18, 27], whereas in women from the Caribbean (Cuba and Puerto Rico), *BRCA2* is more frequently mutated [24, 25]. Studies of women with BC from Spain showed a larger number of rearrangements in both *BRCA* genes when compared to Hispanic women [28–30]. Additionally, several mutations for both *BRCA* genes were found in Hispanic subpopulations, especially in Argentina where there is a higher proportion European admixture. Furthermore, a mutation (*BRCA2* c.156_157insAlu) from Portugal was identified in the Northern/Central region and accounted for one-fourth of the *BRCA1/2* mutations [31]. This same mutation was identified in several families of Brazil [31, 32].

**Table 1:** *BRCA1* and *BRCA2* mutation spectrum in Hispanic populations

			*BRCA1* ^a^			*BRCA2* ^a^		
Study	Population	*n*	Exon	HGVS cDNA	Protein Change	Exon	HGVS cDNA	Protein Change
Guitierrez-Espeleta et al. 2012 [14]	Costa Rica	111	11	c.3403C > T	p.Gln1135*	11	c.5279C > G	p.Ser1760*
						11	c.5303_5304delTT	p.Leu1768Argfs*5
Weitzel JN et al. 2007 [9]	US Hispanics	110	9–12^*^	c.548-?_4185 + ?del (exon deletion)	p.Gly183Alafs*18			
Weitzel JN et al. 2005 [26]	US Hispanics	110	2	c.66_67delAG (Mexico/Spain)	p.Glu23Valfs*17	11	c.2224C > T (Mexico)	p.Gln742*
			11	c.1960A > T (Mexico)	p.Lys654*	9	c.729_732delTGAT (Mexico)	p.Asn243Lysfs*7
			11	c.943ins10 (Mexico)		16	c.7757G > A (Mexico)	p.Trp2586*
			11	c.2864C > A (Mexico/Spain)	p.Ser955_Ser956?fs	11	c.3189_3192delGTCA (Colombia)	p.Ser1064Leufs*12
			11	c.1016dup (Cuba)	p.Val340Glyfs*6	11	c.3264_3265insT (Mexico)	p.Gln1089Serfs*10
			11	c.3598C > T (Mexico)	p.Gln1200*	11	c.4936_4939delGAAA(Guatemala)	p.Glu1646Glnfs*23
			11	c.1086_1141del (Mexico)	p.Asn363Serfs*2	18	c.8322_8323insT (El Salvador)	p. Met2775?Tyrfs*7
			13	c.4327C > T (Mexico/Peru)	p.Arg1443*	23	c.9026_9030delATCAT (El Salvador)	p.Tyr3009Serfs*7
			11	c.2296_2297delAG (Mexico)	p.Ser766*fs			
Weitzel JN et al. 2013 [9]	US Hispanics	746	2	c.66_67delAG	p.Glu23Valfs*17	11	c.3264dupT	p.Gln1089Serfs*10
			9–12^*^	c. 548-?_4185 + ?del (exon deletion)	p.Gly183Alafs*18	3	c.145G > T	p.Glu49*
			5	c.211A > G (Galician founder) (missense)	p.Arg71Gly			
			13	c.4327C > T	p.Arg1443*			
			11	c.3598C > T	p.Gln1200*			
			11	c.2864C > A	p.Ser955*			
John EM et al. 2007 [10]	US Hispanics	393	2	c.66_67delAG	p.Glu23Valfs*17			
			11	c.3029_3030delCT	p.Pro1010Argfs*7			
			11	c.3759_3760delTA	p.Lys1254Glufs*12			
			11	c.4065_4068delTCAA	p.Asn1355Lysfs*10			
			17	c.5035_5039delCTAAT	p.Leu1679Tyrfs*2			
			5	c.211A > G (Galician founder) (missense)	p.Arg71Gly			
Gonzalez-Hormazabal et al. 2010 [16]	Chilean	362	2	c.68_69delAG	p.Glu23Valfs*17	11	c.4742_4743insTG	p.Glu1581Aspfs*37
			11	c.3331_3334delCAAG	p.Gln1111Asnfs*5	11	c.5145_5148delGTAT	p.Tyr1716?Lysfs*8
			11	c.3858_3861delTGAG	p.Ser1286Argfs*20	11	c.6275_6276delTT	p.Leu2092Profs*7
			11	c.4066_4069delCAAG	p.Gln1356Lysfs*9	18	c.8068_8069delGT	p.Val2690Phefs*2
Donenberg T et al. 2010 [22]	Bahamas	214	15	c.4611_4612insG	p.Gln1538Alafs*36		None studied	
			2	c.66_67delAG	p.Glu23Valfs*17			
Delgado L et al. 2011 [76]	Uruguay	111	11	c.2568 T > G	p.Tyr856*	11	c.5351_5352insA	p.Asn1784Lysfs*3
Rodriguez AO et al. 2012 [19]	Colombia	100	11	c.3331_3334delCAAG	p.Gln1111Asnfs*5	11	c.6024_6025insG	p.Gln2009Alafs*9
						11	c.4889C > G	p.Ser1630*
Torres-Mejia et al. 2014 [77]	Mexican	810	9–12	c.548-?_4185 + ?del (exon deletion)	p.Gly183Alafs*8	10	c.1796_1800delTTTAT	p.Ser599*
			11	c.1016dup	p.Val340Glyfs*6	11	c.2808_2811delACAA	p.Ala938Profs*21
			11	c.2296_2297delAG	p.Ser766*fs	11	c.3264_3265insT	p.SGln1089Serfs*10
			11	c.2071delA	p.Arg691Aspfs*10	11	c.6486_6489delACAA	p.Lys2162Asnfs*5
			11	c.3598C > T	p.Gln1200*	11	c.4111C > T	p.Gln1371*
			13	c.4327C > T	p.Arg1443*			
Villareal-Garza et al. 2015 [40]	Mexico	188	9–12^*^	c. 548-?_4185 + ?del (exon deletion)	p.Gly183Alafs*8	11	c.6486_6489delACAA	p.Lys2162Asnfs*5
			11	c.3858_3861delTGAG	p.Ser1286Argfs*20	11	c.6024_6025insG	p.Gln2009Alafs*9
			11	c.4065_4068delTCAA	p.Asn1355Lysfs*10			
			5	c.211A > G (Galician founder) (missense)	p.Arg71Gly			
			11	c.2806_2809delGATA	p.Asp936Serfs*63			
			11	c.3759_3760delTA	p.Lys1254Glufs*12			
			2	c.66_67delAG	p.Glu23Valfs*17			
			13	c.4327C > T	p.Arg1443*			
Ewald IP et al. 2016 [32]	Portugal	145	16–17	c.(4986 + 1_4987-1)_(5074 + 1_5075-1)del (exon deletion)		3	c.156_157insAlu (Founder)	
			19	c.5177_5180delGAAA	p.Arg1726Lysfs*3			
Peixoto A et al. 2009 [31]	Portugal	208				3	c.156_157insAlu (founder)	
Rodriguez RC et al. 2008 [23]	Cuba	307				11	c.3166C > T	p.Gln1056*
						11	c.2376C > A	p.Tyr792*
						11	c.2564_2565delCA	p.Thr855Lysfs*25
Abugattas J et al. 2014 [18]	Peru	266	2	c.66_67delAG	p.Glu23Valfs*17	11	c.2808_2811delACAA	p.Ala938Profs*21
			11	c.1961delA	p.Lys654Serfs*47			
			11	c.3759_3760delTA	p.Lys1254Glufs*12			
Dutil J et al. 2012 [24]	Puerto Rico	23	1-2	c.(?_-48)_(80 + 1_81-1)del (exon deletion)		11C	c.3922G > T (only observed in PR)	p.Glu1308*
						11E	c.5799_5802delCCAA	p.Asn1933Lysfs*29
							c.6393del	p.Lys2131Asnfs*6
						11 F	c.6486_6489delACAA	p.Lys2162Asnfs*5
						15	c.7480C > T	p.Arg2494*
Calderon-Guarcidueñas et al. 2005 [25]	Mexican	22				19	c.8377G > A (missense)	p.Gly2793Arg
Torres D et al. 2007 [20]	Colombia	57	11D	c.3331_3334delCAAG (founder)	p.Gln1111Asnfs*5	11B	c.2808_2811delACAA (founder)	p.Ala938Profs*21
			18	c.5123C > A (founder) (missense)	p.Ala1708Glu	11B	c.5851_5854delGTTA	p.Ser1951Trpfs*11
						11 F	c.6275_6276delTT	p.Leu2092Profs*7
Lara K et al. 2012 [21]	Venezuela	58	11	c.1016dup	p.Val340Glyfs*6	11	c.2808_2811delACCA	p.Ala938Profs*21
			14	c.4603G > T	p.Glu1535*	11	c.3680_3681delTG	p.Leu1227Glnfs*5
Gomes M et al. 2007 [12]	Brazil	402	11C	c.3228_3229delAG	p.Gly1077Alafs*8	11 F	c.6405_6409delCTTAA	p.Asn2135Lysfs*3
			20	c.5263dupC (founder)	p.Glu1756Profs*4			
da Costa ECB et al. 2008 [41]	Brazil	7	20	c.5263dupC (founder)	p.Glu1756Profs*4			
Ewald IP et al. 2011 [37]	Brazil	137	2	c.66_67delAG	p.Glu23Valfs*17	11	c.5722_5723del	p.Leu1908Argfs*2
Dufloth RM et al. 2005 [38]	Brazil	31	20	c.5263dupC (founder)	p.Glu1756Profs*4	11	c.6656C > G	p.Ser2219*
Gallardo M et al. 2006[15]	Chile	77	11D	c.3817C > T	p.Gln1273*	11	c. 5946delT	p.Ser1982Argfs*22
			5	c.189dup	p.Cys64Metfs*2	11	c.6629_6630delAA	p.Glu2210Glyfs*14
			2	c.66_67delAG	p.Glu23Valfs*17	11	c.145G > T	p.Glu49*
Lourenco J et al. 2004 [13]	Brazil	47	20	c.5263dupC (founder)	p.Gln1756Profs*4			
			11D	c.3403C > T	p.Gln1135*			
			11D	c.3331_3334delCAAG	p.Gln1111Asnfs*5			
Solano AR et al. 2012 [17]	Argentina	134	2	c.66_67delAG	p.Glu23Valfs*17	11	c.2808_2811delACAA	p.Ala938Profs*21
			5	c.211A > G (Galician founder) (missense)	p.Arg71Gly	11	c.6037A > T	p.Lys2013*
			7	c.427G > T	p. Glu143*			
			11	c.797_798delTT	p.Ser267Lysfs*19			
			11	c.1504_1507del	p.Leu502Serfs*29			
			11	c.1519delC	p.Arg507Aspfs*25			
			11	c.507_508delAA	p.Arg170Aspfs*11			
			11	c.2686delA	p.Ser896Valfs*104			
			11	c.2728C > T	p. Gln910*			
			11	c.3607C > T	p. Arg1203*			
			11	c.3627insA	p.Glu1210Argfs*8			
			11	c.3758_3759delCT	p. Ser1253*			
			17	c.5030_5033delCTAA	p.Thr1677Ilefs*2			
BIC Database	Portugal		5	c.211A > G (Galician founder) (founder)	p.Arg71Gly	3	c.156_157insAlu	
			11	c.3689 T > G	p.Leu1230*			
Blay P et al. 2013 [28]	Spain (Asturias)	256	2	c.66_67delAG	p. Glu23Valfs*17	3	c.262_263delCT	p.Leu88Alafs*12
			5	c.211A > G (Galician founder) (missense)	p.Arg71Gly	11	c.2830A > T	p.Lys944*
			8	c.470_471delCT	p.Ser157*	11	c.5116_5119delAATA	p.Asn1706Leufs*5
			11	c.1687C > T (Spain founder)	p.Gln563*	11	c.5576_5579delTTAA	p.Ile1859Lysfs*3
			11	c.2900_2901dupCT	p.Pro968Leufs*33	18	c.8042_8043delCA	p.Thr2681Serfs*11
			11	c.3331_3334delCAAG	p.Gln1111Asnfs*5	23	c.9026_9030delATCAT	p.Tyr3009Serfs*7
			11	c.3689 T > G	p.Leu1230*	25	c.9310_9311delAA	p.Lys3104Valfs*6
			11	c.3770_3771delAG	p.Glu1257Glyfs*9			
			11	c.4065_4068delTCAA	p.Asn1355Lysfs*10			
			1–24	c.(−20 + 1_-19-1)_(*1383_?)del (exon deletion)				
			1–13	c.(?_-48)_(4357 + 1_4358-1)del (exon deletion)				
			20	c.5213_5277 + 3182delinsT (exon deletion)				
Fachal L et al. 2014 [29]	Spain (Galicia)	651	1-2	c.(?_-48)_(80 + 1_81-1)del (exon deletion)				
			1–24	c.(−20 + 1_-19-1)_(*1383_?)del (exon deletion)				
			1–13	c.(?_-48)_(4357 + 1_4358-1)del (exon deletion)				
de Juan Jimenez I et al. 2013 [30]	Spain	1763	1-2	c.(?_-48)_(80 + 1_81-1)del (exon deletion)		3	c.145G > T	p.Glu49*
			1-24	c.(−20 + 1_-19-1)_(*1383_?)del (exon deletion)		3	c.262_263delCT	p.Leu88Alafs*12
			20	c.5213_5277 + 3182delinsT (exon deletion)		4	c.370delA	p.Met124Trpfs*12
			2	c.68_69delAG	p.Glu23Valfs*17	10	c.1310_1313delAAGA	p.Lys437Ilefs*22
			5	c.211A > G (Galician founder) (missense)	p.Arg71Gly	11	c.2808_2811delACAA	p.Ala938Profs*21
			11	c.981_982delAT	p.Cys328*	11	c.3170_3174delAGAAA	p.Lys1057Thrfs*8
			11	c.1504_1508delTTAAA	p.Leu502Alafs*2	11	c.3264_3265insT	p.Gln1089Serfs*10
			11	c.1687C > T	p.Gln563*	11	c.3847_3848delGT	p.Val1283Lysfs*2
			11	c.1953_1956delGAAA	p.Lys653Serfs*47	11	c.3922G > T	p.Glu1308*
			11	c.2694dup	p.Val899Serfs*4	11	c.4936_4939delGAAA	p.Glu1646Glnfs*23
			11	c.3257 T > G	p.Leu1086*	11	c.5130_5133delATGT	p.Tyr1710*
			11	c.3331_3334delCAAG	p.Gln1111Asnfs*5	11	c.5576_5579delTTAA	p.Ile1859Lysfs*3
			11	c.3359_3360delTT	p.Val1120Glufs*12	11	c.6275_6276delTT	p.Leu2092Profs*7
			11	c.3583delC	p.His1195Ilefs*15	11	c.6486_6489delACAA	p.Lys2162Asnfs*5
			11	c.3627dup	p.Glu1210Argfs*9	11	c.6629_6630delAA	p.Glu2210Glyfs*14
			11	c.3700_3704del	p.Val1234Glnfs*8	18	c.8042_8043delCA	p.Thr2681Serfs*11
			11	c.3759dup	p.Lys1254*	23	c.9018C > A	p.Tyr3006*
			11	c.3770_3771delAG	p.Glu1257GlyfsX9	23	c.9026_9030delATCAT	p.Tyr3009Serfs*7
			11	c.3785C > A	p.Ser1262*	25	c.9286G > T	p.Glu3096*
			11	c.4065_4068delTCAA	p.Asn1355Lysfs*10			
			12	c.4161_4162delTC	p.Gln1388Glufs*2			
			13	c.4307_4308delCT	p.Ser1436Phefs*4			
			13	c.4357delG	p.Ala1453Glnfs*3			
			14	c.4375A > T	p.Lys1459*			
			15	c.4552C > T	p.Gln1518*			
			16	c.4810C > T	p.Gln1604*			
			17	c.5030_5033delCTAA	p.Thr1677Ilefs*2			
			20	c.5363_5364insC	p.Ala1789Lysfs*41			
			21	c.5311_5333del	p.Pro1771Serfs*51			

^*^According to ClinVar Summary Evidence for this *BRCA1* c.548-?_4185 + ?del mutation is also referenced to as a deletion in exons 8–11. However, in the literature revised was referenced as exon deletion 9–12. Both names are synonymous for the same mutation. Please see https://www.ncbi.nlm.nih.gov/clinvar/variation/219772/#summary-evidence for more information.

^a^Missense mutations pathogenicity status was verified as pathogenic or likely pathogenic by the following databases: ClinVar (https://www.ncbi.nlm.nih.gov/clinvar/) and Invitae (http://clinvitae.invitae.com/).

Several populations have shown the presence of founder mutations, which include some Ashkenazi Jew mutations (*BRCA1* 185delGA; *BRCA1* 5382insC) observed in Mexico [33], Peru [19], and Brazil [13]. These founder mutations have also been reported in Spain [28–30]. Therefore, these Ashkenazi Jew mutations are thought to come from areas of Spain where Jews communities settled during the early Common Era. The *BRCA1* ex9-12 deletion found in Mexican women is also a founder mutation. This mutation has not observed in Spain or South American populations, and was found to pre-date Spanish colonization since it is estimated to have arisen approximately 1,480 years ago [10, 34, 35] (Table 1). Furthermore, two additional founder mutations of Galician and Spanish origin were found in Spain [28]. Of these, the *BRCA1* c.211A > G Galician founder mutation was found in the groups of a US Hispanic and the Argentinian cohort [11, 18]. Three additional founder mutations: 2 in *BRCA1* (*BRCA1* c.3331_3334delCAAG and *BRCA1* c.5123C > A) and 1 in *BRCA2* (*BRCA2* c.2806_2809delACAA) where described in Hispanics from Colombia [21]. These Colombian founder mutations were found in individuals that share the same haplotype, and therefore share a common ancestor [21]. The *BRCA2* c.3922G > T mutation has only been identified in the Puerto Rican cohort, and in US Hispanic studies that include Puerto Ricans and not in other Latin American countries [26]. In Portugal, the *BRCA2* c.156_157insAlu mutation seen in families of Portuguese descent was also identified as a founder mutation that occurred around 558 years ago [31, 32, 36]. Moreover, the *BRCA1* c.5266dup mutation was identified as a founder Brazilian mutation of Eastern European origin [37]. The presence of these founder mutations in Latin America is tightly linked to migration patterns that shaped the genetic background of each Hispanic subpopulation up to present-day.

Several studies suggest that the prevalence of *BRCA* mutations in Hispanic women is higher than the prevalence of *BRCA* mutations among Ashkenazi Jews and non-Hispanic Whites [9, 11]. However, these observations are based on the predictability of *BRCA* genetic risk models that use non-Hispanic White women as reference to determine risk, thus the prevalence of *BRCA* mutations might differ from what has been reported [9]. Differences observed in the *BRCA* mutation spectrum of Hispanic populations could be due to variations in the contribution of each ancestral population to the genetic background of the Hispanic subgroups. In addition, there is very limited information on the mutation spectrum in some countries, such as Costa Rica, Bahama, and Cuba, among others. Therefore, the mutation spectrum of *BRCA* among Hispanic women is still understudied, which may our knowledge on the true percentage of BC in Hispanic populations that may be attributable to hereditary changes.

#### Clinical phenotype

The clinical presentation of HBOC among Hispanic subpopulations is very similar to non-Hispanic cohorts [12, 17, 19, 21, 25, 36–40]. Most studies reported that the majority of women that met inclusion criteria had BC diagnosed before age 50 (67.5%; range: 50.4–81.4%). Additionally, family history of BC was found on an average of 16.1% (range: 5.2–27%) of Hispanic women recruited in the studies described in this review. Comparisons of the age at BC diagnosis among the Hispanic subpopulations described above showed that Caribbean Hispanics (Bahamas, Puerto Rico and Cuba) were diagnosed at later ages (mean: 45.9 years; range: 40–51.8 years) than their South American (mean: 44 years), Central American (mean: 41.6 years) and US Hispanic (mean: 38.5 years) counterparts [17, 19, 21, 25, 28–30, 32, 36–41].

#### Clinical guidelines

The National Comprehensive Cancer Network (NCCN) established guidelines for physicians to evaluate potential candidates for genetic counseling and testing [42]. The evaluation criteria for HBOC genetic testing include: personal history of BC before 50 years of age, one or more 1^st^ degree relatives with BC, family history of a *BRCA1/2* mutation, and personal history of OC, among others. The information obtained from the genetic tests is used by physicians to guide the patient’s medical/surgical management. This management may include chemoprevention therapy, prophylactic surgery (double mastectomy, oophorectomy, and/or hysterectomy), and increased surveillance by mammography and breast MRI. Even though these guidelines have been established, underutilization of these guidelines for Hispanic patients that meet the criteria has been reported [43]. The reason for the low adherence to these guidelines is yet to be addressed, but it is believed that a combination of factors such as, socioeconomic, belief systems, and access to care contribute to this health disparity. Furthermore, the benefits of utilizing these guidelines in other countries, where prevalence of *BRCA* mutations is high, is still to be determined. For example, the high prevalence of *BRCA* mutations in the Bahamas supports the implementation of universal testing. However, countries such as Puerto Rico, that have a small number of mutations, could benefit even more from implementing these guidelines. Additionally, it is important to note that these guidelines where developed using studies mainly performed in non-Hispanic White women. Further studies are needed to elucidate whether NCCN guidelines are useful in Hispanic women or if the differences observed in the prevalence of *BRCA* mutations and clinical presentation, warrants changes to accommodate these differences.

### Colorectal cancer syndromes

Colorectal cancer (CRC) accounts for 11 and 8% of cancers in US Hispanic males and females, respectively [3]. Hereditary CRC accounts for approximately 5–10% of all CRC cases. There are two major types of inherited CRC syndromes: 1) Familial Adenomatous Polyposis (FAP) and 2) Lynch Syndrome (LS). In this section, we will discuss the clinical presentation, genetic basis, and guidelines for testing for these two forms of inherited CRC syndromes.

#### Familial adenomatous polyposis

FAP is characterized by the presence of hundreds to thousands of adenomatous polyps in the colon and rectum. These polyps appear during adolescence and, if left untreated, can develop into CRC by age 40. Affected individuals without a colectomy have close to 100% lifetime risk of developing CRC [44]. By age 20, most patients have at least 1 adenomatous polyp; by age 50, most have developed adenocarcinoma [44]. Thus, the recommended treatment for FAP is prophylactic colectomy in young adulthood [45].

Classic FAP has an incidence ranging from 1 in 8300 people to 1 in 22,000 people, depending on the country of origin [46]. FAP is caused by mutations in the *APC* gene [47]. Most cases have a family history of FAP, but close to 25% of cases are diagnosed as *de novo* mutations [48]. Identifying the specific *APC* mutation in a family can be used for targeted sequencing testing for pre-symptomatic, at-risk family members.

##### Mutation spectrum

The FAP mutation spectrum has been studied in a limited number of Hispanic countries, including Brazil [49], Argentina [50], Peru [51], Mexico [44], Puerto Rico [52], Portugal, and Spain [53] (Table 2). Most mutations identified in Hispanic patients from both Latin America, Portugal, and Spain have been in exon 15 of the *APC* gene (Table 2). This exon comprises more than 75% of the coding sequence and is the most commonly mutated region of the *APC* gene [54].

**Table 2:** *APC* mutation spectrum in Hispanic patients

			*APC* ^a^		
Study	Population	*n*	Exons	Mutation	Protein Change
Cruz-Correa et al. 2013 [43]	Puerto Rico	31	15	c.3183_3187delACAAA	p.Gln1062*
			15	c.4612_4613delGA	p.Glu1538Ilefs*5
			15	c.3149delC	p.Ala1050Glufs*6
			15	c.3927_3931delAAAGA	p.Glu1309Aspfs*4
			14	c.1873C > T	p.Gln625*
			15	c.4012C > T	p.Gln1338*
Tardin-Torrezan G et al. 2013 [40]	Brazil	23		del 5q21.3-q22.3	
			8	c.856_859dupCATG	p.Glu287Alafs*2
			4	c.447dupC	p.Lys150Glnfs*18
			15	c.4097dupC	p.Gln1367Serfs*8
			8	c.904C > T	p.Arg302*
			15	c.4348C > T	p.Arg1450*
			15	c.3880-3881delCA	p.Gln1294Glyfs*6
			8	c.847C > T	p.Arg283*
			15	c.4099C > T	p.Gln1367*
			15	c.3050-3053delATGA	p.Asn1017Metfs*4
			15	c.3927-3931delAAAGA	p.Glu1309Aspfs*4
			15	c.4393-4394delAG	p.Ser1465Trpfs*3
Zeichner S et al. 2012 [41]	Hispanic	1	15	c.3927_3931delAAAGA	p.Glu1309Aspfs*4
Ricker C et al. 2010 [42]	Mexico, Guatemala, Honduras	9	15	c.3184C > T	p.Gln1062*
			15	c.3709delCA (MX)	p.Gln1237Glufs*2
				g.89926-?_141606 + ?del (GU)	
			15	c.3927_3931delAAAGA (MX)	p.Glu1309Aspfs*4
			15	c.3803_3815del1 (HO)	p.Pro1268Glufs*16
			15	c.3183_3187delACAAA (MX)	p.Gln1062*
InSIGHT [53]	Argentina		15	c.3920 T > A (missense)	p.Ile1307Lys
	Portugal		15	c.4687dup	p.Leu1563Profs*4
			15	c.5826_5829delCAGA	p.Asp1942Glufs*27
	Spain		5	c.637C > T	p.Arg213*
			6	c.707delA	p.Gln236Argfs*57
			7	c.730_731delAG	p.Arg244Valfs*7
			7	c.802G > T	p.Glu268*
			8	c.858delT	p.His286Glnfs*7
			8	c.904C > T	p.Arg302*
			11	c.1412delG	p.Gly471Aspfs*27
			13	c.1682dupA	p.Thr562Aspfs*19
			13	c.1690C > T	p.Arg564*
			14	c.1787C > G	p.Ser596*
			14	c.1946_1947insG	p.Asn649Lysfs*2
			15A	c.1993_1994delTT	p.Leu665Ilefs*9
			15	c.2310delA	p.Glu771Lysfs*6
			15B	c.2413C > T	p.Arg805*
			15C	c.2496delC	p.Ser833Alafs*9
			15D	c.2838_2839delAT	p.Cys947Phefs*15
			15D	c.2977A > T	p.Lys993*
			15D	c.2980_2990del11	p.Phe994Trpfs*10
			15E	c.3224dupA	p.Tyr1075*
			15E	c.3251_3252dupAT	p.Lys1085Ilefs*42
			15G	c.3921_3924delAAAA	p.Ile1307Metfs*13
			15G	c.3927_3931delAAAGA	p.Glu1309Aspfs*4
			15	c.4260_4261delCA	p.Ser1421*

^a^Missense mutations pathogenicity status was verified as pathogenic or likely pathogenic by the following databases: ClinVar (https://www.ncbi.nlm.nih.gov/clinvar/) and Invitae (http://clinvitae.invitae.com/)

##### Clinical phenotype

The data available on the clinical manifestation of FAP in Hispanic is limited. Of the studies discussed, only studies from Puerto Rico [52], US Hispanics (from Mexico, Guatemala and Honduras) [51] and Brazil [49] had phenotype information for Hispanic FAP patients. Puerto Rican individuals with FAP had a mean age of diagnosis of 27.6 years (range: 9–71 years), the majority were male (51.6%), and had ≥ 100 polyps in their colon (67.7%) [52]. In addition, upper gastrointestinal polyps and desmoid tumors were observed in 41.9 and 9.7% of the study subjects, respectively. Ricker et al. described 9 Hispanic women from different regions with FAP with a mean age of diagnosis of 37.2 years. Among these women the most common extracolonic manifestation as upper gastric polyps [51]. The study describing Brazilian FAP patients reported that subjects had a mean age of 32.6 years (7–67 years), 79% had upper gastrointestinal polyps, and 54% had desmoid tumors [49]. The prevalence of extracolonic manifestations in the FAP patients of the Brazilian cohort was higher than in other Hispanic subpopulations. In summary, the clinical manifestations of FAP in Hispanics are similar to the one presented by non-Hispanic US Whites.

##### Clinical guidelines

To guide genetic testing for FAP in individuals at high-risk, both the NCCN and the American Gastroenterology Association (AGA) have developed guidelines for physicians. The NCCN guidelines recommend genetic counseling for individuals with a personal history of ≥20 colorectal adenomas, known *APC* deleterious mutation in the family, and personal manifestation of a desmoid tumor, cribriformular variant of papillary thyroid cancer, or hepatoblastoma [55]. Guidelines from the AGA for genetic counseling and testing include, personal history of >10 cumulative colorectal adenomas, family history of FAP, and family history of FAP-type extracolonic manifestations [56].

#### Lynch Syndrome (LS)

LS accounts for 2–4% of all CRC cases [44]. This syndrome is characterized by CRC at an early age and a high risk for a number of additional primary cancers, including endometrial and gastric cancer [57]. Approximately 1 in 35 individuals diagnosed with CRC has LS [58]. During their lifetime, individuals with LS will have up to a 70% risk of developing CRC [58]; women will have up to a 45% risk of developing endometrial cancer [59].

##### Mutation spectrum

Germline mutations in the DNA mismatch repair (MMR) genes, *MLH1*, *MSH2*, *MSH6*, *PMS2* and *EPCAM,* causing the absence of MMR protein expression result in LS [60]. The most common genes mutated in LS are *MLH1* and *MSH2*; close to 50% of LS individuals have a mutation in *MLH1* [61]. In a review published by Dominguez-Valentín et al., the LS mutation spectrum in South American countries was described [62]. Pathogenic mutations in the MMR genes were identified in patients from Brazil [53], Argentina [63], Uruguay [64], and Colombia [65] (Table 3). Furthermore, the InSIGHT database contains additional information on MMR mutations in additional Hispanic subpopulations, Portugal, and Spain [53]. As seen in non Hispanic Whites, mutations in the *MLH1* gene are more common in Hispanic, Portuguese and Spanish populations, with the exception of Uruguay [64] and Caribbean Hispanics (Puerto Rico) [66] (Table 3). Data from Caribbean Hispanic patients showed that the mutation spectrum consisted mostly of *MSH2* mutations (66.7%), followed by *MLH1* mutations (25.0%) [66]. Mutations in the *MSH6* gene have only been identified in the Caribbean Hispanics [66], Brazilians [53], Chileans [67], and Cubans [53]. There are no published mutations in the *EPCAM* gene among Hispanics cohorts. The mutation spectrum of MMR genes in Hispanic subgroups could be more varied, thus, additional studies in Hispanic subpopulations are needed.

**Table 3:** *MMR* genes mutation spectrum in Hispanic patients

			*MLH1* ^a^			*MSH2* ^a^		
Study	Population	n	Exon	Mutation	Protein Change	Exon	Mutation	Protein Change
Cruz-Correa et al. 2015 [57]	Puerto Rico	89	11	c.1024del16	p.Met342Cysfs*25	5	c.905 T > A	p.Leu302*
			18	c.2044_2045delAT	p.Met682Valfs*11	11	c.1705delGA	p.Glu569Ilefs*2
			16	c.1855delG	p.Ala619Leufs*18	9	c.1457del4	p.Asn486Thrfs*10
						1–3	c.(?_-68)_645 + ?del (exon deletion)	
Giraldo et al. 2005 [56]	Colombia	11	16	c.1855delG	p.Ala619Leufs*18	3	c.596delG	p.Cys199Leufs*15
Wielandt et al. 2012 [58]	Chile	35 fam	14 to 15	c.1559-?_1731 + ?del	p.Val520_Ser577 > Gfs*7	5	c.897 T > G	p.Tyr299*
			19	c.2104-?_2271 + ?del	p.Ser702_X757del			
Sarroca et al. 2003 [55]	Uruguay	461				1	c.181C > T	p.Gln61*
						3	c.530_531delAA	p.Glu177Valfs*3
Vaccaro et al. 2007 [54]	Argentina	306	16	c.1852_1854del	p.Leu618del	12	c.1911del	p.Arg638Glyfs*47
						3	c.388_389del	p.Asn130Valfs*2
InSIGHT [53]	Argentina		8	c.677G > A (missense)	p.Arg226Gln	1	c.166G > T	p.Glu56*
			16	c.1890dup	p.Asp631*	3	c.388_389del	p.Gln130Valfs*2
						7	c.1224 T > A	p.Tyr408*
						13	c.2046_2047del	p.Val684Aspfs*14
	Brazil		2	c.175dup	p.Ile59Asnfs*20	1	c.187del	p.Val63*
			8	c.677G > A (missense)	p.Arg226Gln	5 to 6	c.793-?_1076 + ?del	p.Val265Ilefs*29
			9	c.779 T > G (missense)	p.Leu206Arg	7	c.1249del	p.Val417Leufs*21
			12	c.1276C > T	p.Gln426*	9	c.1444A > T	p.Arg482*
			13	c.1459C > T	p.Arg487*	9	c.1447G > T	p.Glu483*
			14	c.1639_1643dup	p.Leu549Tyrfs*44	11	c.1667del	p.Leu556*
			16	c.1853delinsTTCTT	p.Lys618Ilefs*4	11	c.1738_1741delGAAAA	p.Glu580Leufs*9
			17	c.1975C > T	p.Arg659*	12	c.1967_1970dup	p.Asp660Glufs*3
			18	c.2041G > A (missense)	p.Ala681Thr	13	c.2131C > T	p.Arg711*
			19	c.2224C > T	p.Gln742*	13	c.2152C > T	p.Gln718*
						15	c.2525_2526del	p.Glu842Valfs*3
						16	c.2785C > T	p.Arg929*
	Puerto Rico		10	c.866_867dup	p.Pro290Thrfs*8			
	Uruguay		8	c.665del	p.Asn222Metfs*7	1	c.181C > T	p.Gln61*
						3	c.530_531	p.Glu177Valfs*3
	Portugal		10	c.793C > T (missense)	p.Arg265Cys			
	Spain		2	c.155_158del	p.Lys52ArgfsX4	3–6	c.224-?_1003 + ?del	p.Ala123Ilefs*2
			2	c.199G > A (missense)	p.Gly67Arg	4	c.761del	p.Asn254Ilefs*20
			4	c.332C > T (missense)	p.Ala111Val	4	c.691delG	p.Asp231Thrfs*15
			4	c.350C > T (missense)	p.Thr117Met	6	c.1035G > A	p.Trp345*
			5	c.382del	p.Ala128Glnfs*8	7	c.1249_1253del	p.Val417Thrfs*7
			8	c.665del	p.Asn222Metfs*7	7	c.1255C > T	p.Gln419*
			8	c.677G > A (missense)	p.Arg226Gln	9	c.1399G > T	p.Glu467*
			9	c.701delA	p.Glu234GlyfsX4			
			9	c.731G > A (missense)	p.Gly244Asp			
			13	c.1420del	p.Arg747Glyfs*17			
			13	c.1459C > T	p.Arg487*			
			16	c.1865 T > A (missense)	p.Leu622His			
			16	c.1893del	p.Asp631Glufs*6			

^a^Missense mutations pathogenicity status was verified as pathogenic or likely pathogenic by the following databases: ClinVar (https://www.ncbi.nlm.nih.gov/clinvar/) and Invitae (http://clinvitae.invitae.com/)

##### Clinical presentation

For *MLH1* mutation carriers, the CRC lifetime risk has been shown to be as high as 68% [68]. The prevalence of *MSH2* mutations in families with LS has been reported to range from 38 to 54%, with a CRC lifetime risk of up to 68% [69]. The clinical presentation of LS in the Hispanic subgroups, is similar to what has been observed in NHW with a mean age of diagnosis of 30–44 years of age, endometrial cancer being the 2^nd^ most common LS-associated cancer, and colorectal tumor predominantly in the right colon [62].

##### Clinical guidelines

The identification of at-risk individuals for LS includes the clinical profile of the patient and molecular testing. The NCCN has published clinical guidelines to determine a patient’s risk of having LS [55]. The Amsterdam and Bethesda criteria were established to help identify individuals that may harbor germline mutations in the MMR genes based on personal and family history of CRC [70]. The American Gastroenterology Association (AGA) also provides guidelines for the identifying at risk individuals and additionally guidelines for the screening of CRC among these LS individuals [71]. The AGA guidelines for LS testing are: to test MMR deficiency in newly diagnosed CRC cases, and to test individuals with CRC diagnosed at age ≤70 or individuals older than 70 years of age who have a family history of LS [71]. Moreover, the AGA guidelines for screening for LS in these high-risk individuals or those affected by LS suggest colonoscopies every 1 to 2 years starting at age 20–25 or starting 2 years younger than the youngest age at CRC diagnosis in the family [71]. Additional studies are needed to determine the utilization of established guidelines for Lynch Syndrome in Hispanics.

In addition, assessment of molecular markers in tumors, such as microsatellite instability (MSI) or loss of MMR protein expression, are used as molecular screening tools for identifying LS patients. Loss of MMR protein expression correlates with the presence of MSI and is indicative of a possible germline MMR gene mutation [72]. Genetic testing for LS is considered the “gold standard” for diagnosis. However, lack of detection of MMR protein expression by immunohistochemistry (IHC) can be a surrogate marker to diagnose LS when genetic testing is not available, due to limited accessibility to this type of test (lack of insurance coverage, economic hardships, limited infrastructure). Current NCCN and AGA guidelines recommend that genetic testing be offered to at-risk patients if the test influences the medical management of their condition or that of their at-risk relatives. However, an important concern is that these criteria may miss LS patients who do not meet criteria. LS screening of all CRC tumors has been shown to be cost-effective and is currently recommended by multiple professional societies [55, 71]. As many as 1/3 of women with LS may initially present with endometrial cancer without a history of CRC, thus universal screening for LS in endometrial cancer has also been proposed [73]. Currently, there are no reports in the literature on universal testing of Lynch Syndrome in Hispanic subgroups using IHC detection of MMR proteins, in contrast with non-Hispanic whites for which data is abundant.

### Multi-gene panel testing

The Supreme Court of the US invalidated the patents made by Myriad Genetics© that gave them the right to exclusively offer *BRCA* genetic testing. This ruling permitted additional methods to sequence the *BRCA* gene, as well as other genes, thus significantly reducing costs for genetic testing. Furthermore, major breakthroughs in next-generation sequencing (NGS) and a more comprehensive understanding of the genetic basis underlying hereditary cancers are shifting genetic testing for hereditary cancer from single gene testing to multiple gene panels. Several of the commercial companies offering cancer genetic screening are offering panels comprised of dozens of genes, including *BRCA1*/*2* and the MMR genes, among others. Multi-gene testing would be especially beneficial to families who present a spectrum of different cancer types. Furthermore, the current reduced cost of NGS technology will allow laboratories across Latin America to perform studies on patients with the cancer syndromes discussed above to continue understanding the mutation spectrum and prevalence of mutations of Hispanic populations.

Although the future of hereditary cancer testing will undoubtedly reside in multi-gene panel testing, it presents some important challenges. Multi-gene panels include moderate penetrance genes with limited clinical utility. Furthermore, for many of the known hereditary cancer genes, is not clear whether the identification of a deleterious mutation warrants clinical measures beyond increased surveillance, particularly among understudied ethnic groups such as Hispanics [74]. In addition, multi-gene panel testing is likely to result in the identification of a higher number of variants of uncertain significance (VUS) and mutations with an undetermined clinical value. Currently, a lack of standardization across genetic laboratories regarding the analysis of the data as well as the clinical interpretation of results may lead to erroneous medical advice [75]. As research, education, and health policy development advances, these issues are likely to be resolved.

## Conclusion

Over the last 10 years, the scientific community has conducted research to determine the prevalence and mutation spectrum of the genes associated with hereditary cancer syndromes in Hispanic populations. Genetic testing is an integral part of the treatment for cancer patients. As the standard of care in hereditary cancer testing shifts from single gene testing to multi-gene panel testing, it will be essential that ongoing research efforts focus on determining mutation penetrance and associated genetic risks. This information will ensure that the standard-of-care genetic testing is provided to all patients in need and that patients will have access to the latest advances in prevention and treatment of hereditary cancers. Examination of barriers to genetic testing and implementation of culturally sensitive educational programs will be essential to increase adherence. Moreover, integration of clinical genetic guidelines to health policy will enable implementation and sustainability of genetic medicine across Hispanic populations.
